# TIHM: An open dataset for remote healthcare monitoring in dementia

**DOI:** 10.1038/s41597-023-02519-y

**Published:** 2023-09-09

**Authors:** Francesca Palermo, Yu Chen, Alexander Capstick, Nan Fletcher-Loyd, Chloe Walsh, Samaneh Kouchaki, Jessica True, Olga Balazikova, Eyal Soreq, Gregory Scott, Helen Rostill, Ramin Nilforooshan, Payam Barnaghi

**Affiliations:** 1 Imperial College London, Department of Brain Sciences, London, W12 0NN UK; 2 The UK Dementia Research Institute, Care Research and Technology Centre, London, W1T 7NF UK; 3 Surrey and Borders Partnership NHS Trust, Leatherhead, KT22 7AD UK; 4 University of Surrey, Guildford, GU2 7XH UK

**Keywords:** Translational research, Dementia

## Abstract

Dementia is a progressive condition that affects cognitive and functional abilities. There is a need for reliable and continuous health monitoring of People Living with Dementia (PLWD) to improve their quality of life and support their independent living. Healthcare services often focus on addressing and treating already established health conditions that affect PLWD. Managing these conditions continuously can inform better decision-making earlier for higher-quality care management for PLWD. The Technology Integrated Health Management (TIHM) project developed a new digital platform to routinely collect longitudinal, observational, and measurement data, within the home and apply machine learning and analytical models for the detection and prediction of adverse health events affecting the well-being of PLWD. This work describes the TIHM dataset collected during the second phase (i.e., feasibility study) of the TIHM project. The data was collected from homes of 56 PLWD and associated with events and clinical observations (daily activity, physiological monitoring, and labels for health-related conditions). The study recorded an average of 50 days of data per participant, totalling 2803 days.

## Background & Summary

Dementia is most commonly characterised by symptoms of cognitive decline, such as memory loss and problems with attention; however, up to 90% of PLWD will also experience behavioural and psychological symptoms, including sleep disturbances, agitation, and apathy^1^. In addition, up to approximately 1 out of 4 unplanned hospital admissions for PLWD are due to potentially preventable causes such as severe UTI, falls, and respiratory problems. These symptoms and events affect the health and well-being of PLWD, increase the stress and anxiety of caregivers, and increase the demand on healthcare services. As such, providing timely and effective interventions is a significant challenge in dementia care and requires frequent, reliable, and privacy-aware activity and health monitoring for PLWD.

The TIHM project employs low-cost, Internet of Things (IoT) sensing technologies to enable predictive and proactive in-home healthcare monitoring. By supporting the integration of analytical solutions, the TIHM platform allows us to develop clinically applicable machine intelligence and decision-support methods for early and personalised interventional care.

Within TIHM, remote devices for collecting vital signs, and environmental and activity data were used to monitor the day-to-day well-being of PLWD^2,3^. The use of these technologies can help PLWD retain their independence for longer periods of time and provide caregivers with evidence-based information that may reduce potential anxiety and depression in PLWD^4^. Furthermore, the integration of machine learning methods and in-home monitoring technologies allows for the identification of changes in cognition and physical well-being. Several studies have applied machine learning and analytical techniques to the data collected as part of the TIHM project to investigate activity and health patterns and develop methods to detect and predict conditions that affect the wellbeing of PLWD and caregivers^5,6^.

A major issue with current remote monitoring systems is the heterogeneity of the underlying devices and technologies^7,8^. Different devices use different data formats and proprietary interfaces and applications to present the data, making it difficult to integrate information from various sources and process them in (near-) real-time. These conflicting setups hinder the process of extracting patterns, detecting anomalies, and performing predictive analysis using integrated data from different digital sources. TIHM provides an integration of data from various sources and modalities to transform in-home monitoring applications and create intelligent decision-making support systems using routinely collected data. By applying machine learning models that are designed with partially labelled, multi-modal, noisy and dynamic data in mind, we have developed several explainable methods for detecting and predicting adverse health conditions and events^6,9^.

In this paper, we present the TIHM dataset^10^ collected during the feasibility study phase of the project. This includes anonymised information on daily activities, sleep monitoring, clinical and physiological data, and corresponding labelled health events. The dataset collected through the TIHM project can be employed for studies that develop analytical and machine learning solutions for continuous healthcare monitoring, especially in dementia care. It can offer preliminary data to design and validate methods that analyse multi-modal data with sparse annotations for healthcare monitoring applications. For example, new AI methods could be developed to detect: i) vital sign abnormalities; ii) neuropsychiatric symptoms; iii) social isolation; and iv) functional decline.

## Methods

A secure digital platform was developed to integrate in-home IoT and remote monitoring technologies to collect routine physiological, sleep, movement, and ambient data^11^.

### Digital markers

Digital markers are measurable physiological and in-home movement data gathered and assessed by digital devices, including portable and passive monitoring sensors. Digital markers can deliver novel and useful insights into an individual’s activity patterns and physiological health, allowing for continuous and non-invasive healthcare monitoring. Remote monitoring technologies also provide a novel approach to monitoring the effect of new interventions in clinical trials and observational and interventional studies^12^.

**Fig. 1:**
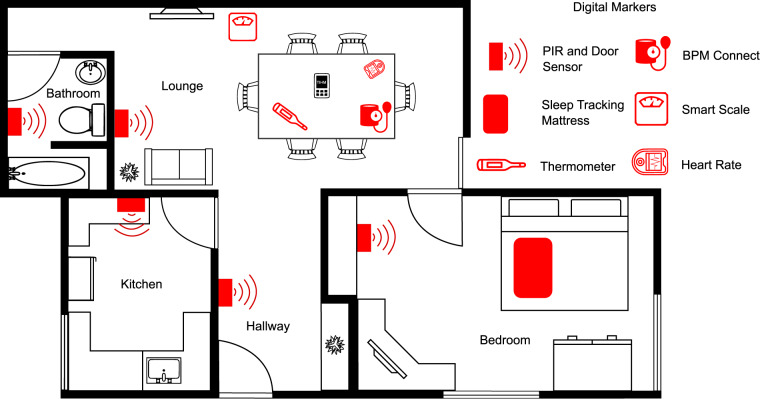
Demonstration of a residential setting equipped with PIR sensors for in-home activity monitoring and other sensors for sleep and physiology monitoring in the TIHM project. PIR and door sensors are included in each room of the house. An under-the-mattress sensor is used for sleep and in-out-of-bed monitoring. Connected devices which are operated manually are also used in the setting to acquire physiology data.

In TIHM, sensory devices were installed in participants’ homes, and activity data was continuously recorded via passive infrared (PIR) sensors (installed in the hallway and living room), movement sensors (on kitchen, bedroom and bathroom doors), door sensor (installed in the main entrance), and an under-the-mattress sleep-mat (for monitoring sleep and in-out of bed activity). Participants were supplied Bluetooth-enabled devices to measure their blood pressure, heart rate, body temperature, weight, and hydration daily. Figure 1 shows an example of the residential setting of a participant in the study equipped with the sensors. Details of the devices and digital markers are shown in Tables 1, 2.

**Table 1:** Overview of the digital markers collected in the TIHM dataset^10^, detailing the monitoring device used and the frequency of measurement for the collection of data.

Digital Marker	Monitoring Device	Frequency
Sleep	Sleep Tracking Mat	Every minute
In-home movement	PIR and Door Sensors	Continuous from multiple locations (precision in seconds)
Body/Skin Temperature	Thermometer	Continuous in a short period per day (precision in seconds)
Diastolic/Systolic Blood Pressure	BPM Connect	Once a day
Heart Rate	Heart Rate	Once a day
Muscle Mass/Body Weight/Total Body Water	Smart Scale	Once a day

**Table 2:** List of devices used for data collection in the study, including manufacturer, device type, and specific product model with links to specifications.

Device Type	Manufacturer	Specification
PIR Sensor	Develco Products - Motion Sensor Mini	Radio Sensitivity: −92 dBm; Output power: +3 dBm; Sensitivity range: 9 m/30 ft
Door Sensor	Develco Products - Window Sensor	Radio Sensitivity: −98 dBm; Output power: +8 dBm; Magnetic: 0.1–1.0 cm
Heart Rate	iHealth Labs - Air Pulse Oximeter	SpO2 measuring range: 70–99%; SpO2 accuracy: ±2%; Pulse rate measuring range: 30–250 bpm; Pulse rate accuracy: ±2 bpm
Weight Scale & Body Composition	iHealth Labs - Core Body Composition Scale	Body weight range: 11 lb-400 lb/5 kg-180 kg; Body fat range: 5.0%–65.0%; Body weight range: ±1.1 lb/0.5 kg(11–110 lb/5 kg-50 kg), ±1% (110–400 lb/50 kg–180 kg); Body fat accuracy: ±1%
Blood Pressure	iHealth Labs - Clear Wireless Blood Pressure Monitor	Systolic range: 60–260 mmHg; Diastolic range: 40–199 mmHg; Pulse rate range: 40–180 bts/min. Pressure accuracy: ±3 mmHg; Pulse rate accuracy: ±5%
Thermometer	Withings - Smart Temporal Thermometer	Clinical accuracy: ±0.2 °C; Temperature range: 35 °C - 43.2 °C; Resolution: 0.1 °C
Sleep Tracking Mat	Withings - Sleep Analyzer	Sleep duration, sleep onset and time to wake; Sleep cycles: deep, light, REM phases; Continuous and average heart-rate; Snoring duration

### Participants

To be eligible for this study, participants needed to meet the inclusion criteria of being a person over 50 years old, with a verified diagnosis of dementia (of any type) or mild cognitive impairment, who has the capacity to provide informed consent to participate in the study, and either received treatment from an Old Age Psychiatry department in the past or is currently on their caseload. In addition, participants required a study partner or caregiver who had known the PLWD for at least six months and was able to attend research assessments with them. If a participant was unable to provide information about their health, their partner or caregiver completed the necessary assessments on their behalf. Individuals with unstable mental states, including severe depression, severe psychosis, agitation, anxiety, active suicidal thoughts, or those receiving treatment for terminal illnesses were not included in the study. A total of 56 people were selected as participants. All the participants have granted the publication of this dataset. The demographic details of the participants in the dataset is shown in Table 3. Some participants in the study requested not to share all or part of their information outside the study. For these cases, the corresponding information is represented by “N/A” (Not Available) in Table 3 and their data was not included in the dataset.

**Table 3:** Demographics of the participants in the study (n = 56).

**Sex, n = 56**	
Female	28
Male	28

**Age, n = 56**	
70–80	17
80–90	26
90–100	13

**Ethnicity, n = 56**	
White	48
Other	3
N/A	5

**Household, n = 56**	
Lives Alone	14
Lives with Partner	41
N/A	1

**Dementia Diagnosis, n = 56**	
Alzheimer’s Disease	40
Vascular Dementia	6
Other	9
N/A	1

Participants who requested not to disclose their information have been represented by “N/A” (Not Available). For smaller sub-groups (n < 5), to avoid any privacy risks, we have changed the information to N/A in the dataset.

### Ethical approval

The TIHM study received ethical approval from the London-Surrey Borders Research Ethics Committee; TIHM 1.5 REC: 19/LO/0102. The study is registered with National Institute for Health and Care Research (NIHR) in the United Kingdom under Integrated Research Application System (IRAS) registration number 257561. To the best of our knowledge, this is the first publicly available dataset for remote healthcare monitoring for PLWD that includes in-home activity and sleep data, physiological measurements, and labelled health and care-related events during the monitoring period. TIHM is also currently being offered as a service by the Surrey and Borders National Health Service (NHS) Trust in the United Kingdom.

### Dataset collection

We combined in-home sensory data with individuals’ healthcare information extracted from General Practitioner (GP) records and hospital visits to create a holistic view of their well-being and care needs.

The sensor deployment relied on off-the-shelf devices to monitor in-home activities and physiology. These sensors continuously collected and communicated the data to a data collection and integration platform. The data from the sensors in this release are de-identified, cleaned (removing redundant and multiple records) and merged based on their categories into four different tables which are further explained in Section Data Records. The annotations and data labels for this study were collected by a monitoring team who contacted the participants to determine if they had experienced a health-related event. The data was labelled as true if the monitoring team validated the presence of a health-related event and false if there was no event.

The initial alert generation for triaging a healthcare event was governed by a set of rules and thresholds applied to physiological measurements and the output of an analytical model designed to analyse in-home activity and physiology data^13^. This initial analytical model was only intended to guide the monitoring team in identifying episodes of agitation and creating a labelled dataset for further data analysis and machine learning developments.

By combining the data from the in-home sensors, we obtained a comprehensive understanding of an individual’s home activity and health, and used this information to determine the risk or presence of health related conditions^9^. For example, we detected changes in an individual’s activity patterns, such as a change in room usage that may indicate social isolation or agitation^5^.

### Dataset de-identification

Two types of de-identification have been applied to data. During the study, the data was pseudo-anonymised for the clinical monitoring team and for developing analytical models. The data includes the demographics (age and sex) in addition to raw sensory observations and measurements. Information governance and control methods and procedures were applied to the data during the project. An NHS-approved Data Processing and Impact Assessment was conducted for the data collection, storage and access procedures. Before making the TIHM dataset^10^ available online, the data was then fully anonymised by removing all personally identifying information or identifiable attributes. Participants are randomly assigned with a universally unique identifier (UUID) to increase security in the de-identification.

## Data Records

The TIHM dataset^10^ is available at Zenodo. It consists of five separate tables (Activity, Sleep, Physiology, Labels, and Demographics) containing information about various aspects of remote healthcare monitoring. A description of the data files included in the TIHM dataset^10^ is shown in Table 4. Each table includes timestamps related to each event and the assigned UUIDs of the participants to allow for cross-referencing and synchronisation among the various records.

1. The ***Activity*** table includes data from motion and door sensors that track movement in different locations in the home. The temporal resolution for this data is in seconds. For each recorded activity, the locations may be a subset of the commonly recorded locations in the home, which include ‘Back Door’, ‘Fridge Door’, ‘Hallway’, ‘Kitchen’, ‘Lounge’, ‘Bedroom’, Bathroom’, ‘Front Door’, and ‘Dining Room’.
2. The ***Sleep*** table includes sleep data collected using sleep tracking mats. This data includes four sleep states (i.e., awake, light, deep, REM), as well as information on snoring, heart rate, and respiratory rate reported by the sleep-mat device. The temporal resolution of the heart rate, breathing rate, and sleep state data is per minute, whilst a PLWD is in bed, on top of the device.
3. The ***Physiology*** table contains daily records of vital signs, including body temperature, skin temperature, diastolic blood pressure, systolic blood pressure, heart rate, muscle mass, body water, and body weight. Some participants may not have recorded this information on a daily basis, resulting in sparsity in the physiology data.
4. The ***Labels*** table includes data on six types of alerts that have been verified by the monitoring team in the TIHM study. These labels include episodes of agitation, abnormally high or low blood pressure, abnormally high or low body temperature, low body water (i.e. dehydration), abnormally high or low heart rate, and weight changes. Seven participants did not have any confirmed alerts during the project and are not included in this table. The *Labels* table can be used for training predictive models. The thresholds used to raise and verify these alerts are shown in Table 5.

**Table 4:** An overview of the data files included in the TIHM dataset^10^.

Table Name	Description
Activity	Activation of the movement sensors in multiple locations for 56 participants.
Sleep	Sleeping data collected by sleep tracking mat for 17 participants, including four sleep states (awake, light, deep, REM), snoring or not, heart rate, and respiratory rate.
Physiology	Physiology records for 55 participants, including eight types of physiological measurements.
Labels	Clinically verified alerts for 49 participants, including six types of alerts: agitation, blood pressure, body temperature, body water, pulse, and weight.
Demographics	Demographic information for 56 participants, including sex and age group of each participant. There are three age groups in total: (70,80], (80,90], (90,110].

All data files are in Comma Separated Values (*CSV*) format.

**Table 5:** List of health-related events alerts that are generated based on the measurements of different health parameters and their respective thresholds.

Alert	Thresholds	Time algorithm runs
Blood Pressure (mmHg)	Systolic: *x* < 80, *x* > 190 Diastolic: *x* < 50, *x* > 110	Measured daily. Alert raised if one or both systolic and diastolic blood pressures are out of the threshold values
Pulse (BPM)	*x* < 55, *x* > 100	Measured daily. Alert raised with the severity of excess.
Body Temperature ()	*x* < 35, *x* > 37.6	Measured daily. Alert raised with the severity of excess.
Body Water (%)	*x* < 40, *x* > 70	Measured daily. Alert raised with the severity of excess.
Body Weight (Kg)	5% loss of: Week, Month, Two Months, Three Months	Measured daily. Alert raised if weight value more than 5% less than the average in a measurement period. The smallest period has higher precedence.
Agitation	Episodes of agitation in a 6-hour period.	Alert generated every 6 hours based on the monitoring data processed by an analytical model.
5. The ***Demographics*** table provides sex and age group information for each participant. All participants are separated into three age groups: (70, 80], (80, 90], (90, 110].

**Fig. 2:**
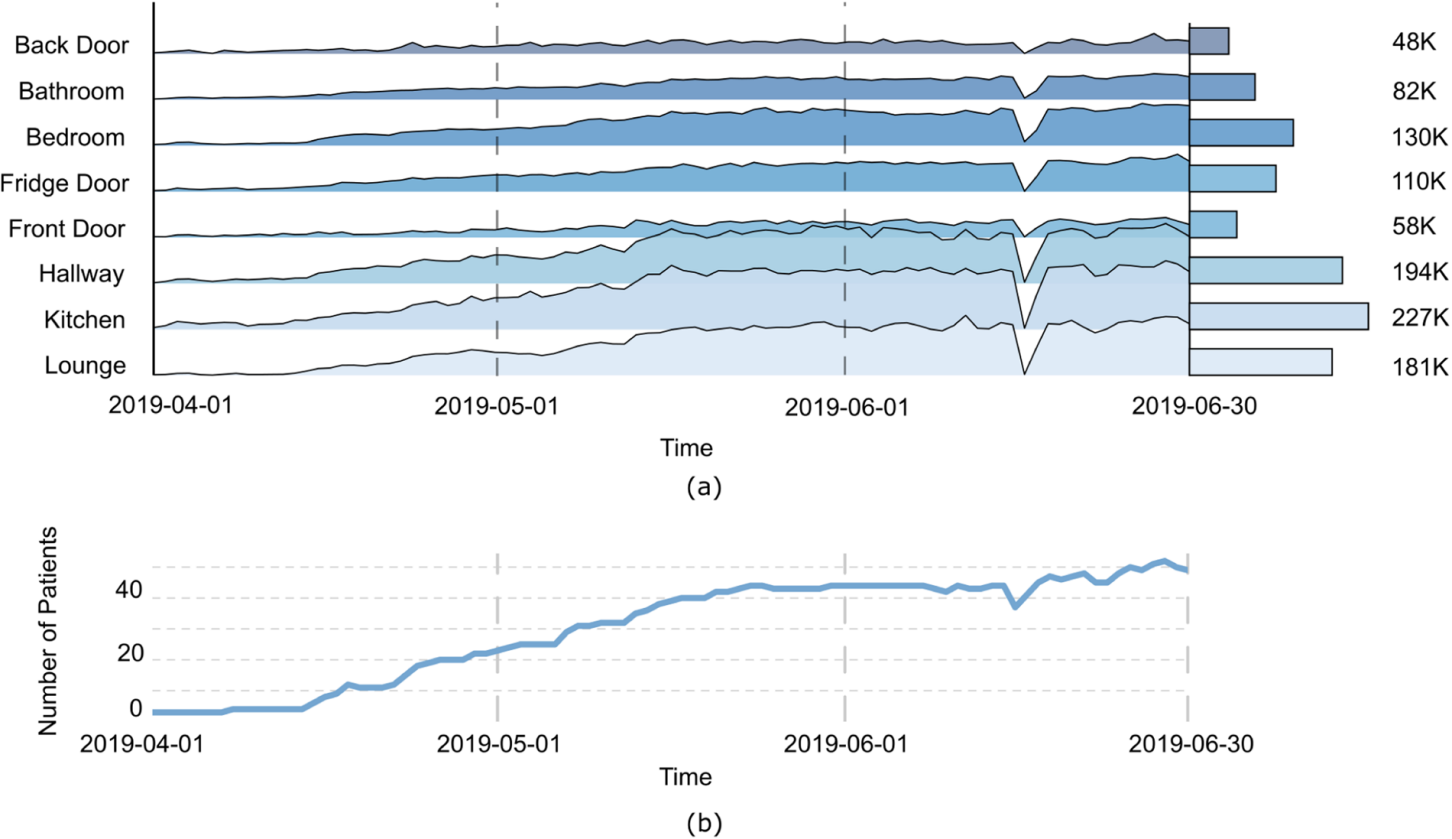
(**a**) An overview of the number of movements per location, per day. The bar chart on the right shows the total number of movements that occurred at each location over the given period. The large drop on the 14th of June 2019 is caused by a technical failure in the data collection server. (**b**) An overview of the total number of participants joining the study within the timeline, the increasing trend of activities (**a**) corresponds to an increase in the number of households and participants recruited (**b**). On average, each participant was involved in the study for about 50 days.

An overview of the *Activity* table is shown in Fig. 2, which summarises total in-home movement in each location daily for all the participants. The increasing trend of the total number of in-home movements aligns with the increasing number of participants in the study. The figure also shows a large drop on the 14th of June 2019, which was caused by a technical failure in the data collection server. A similar phenomenon can also be observed in other tables.

**Fig. 3:**
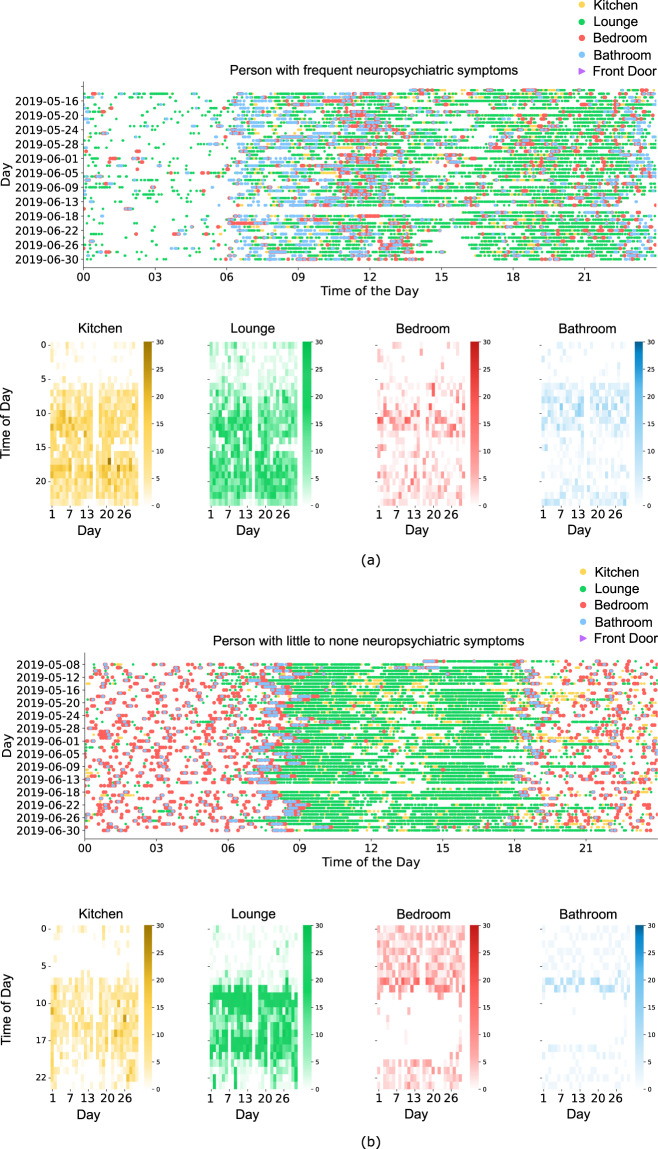
(**a**) In-home activities of PLWD with frequent episodes of neuropsychiatric symptoms. Activity patterns are irregular and there are no consistent habitual patterns of daily activities. (**b**) In-home activities of a PLWD with no neuropsychiatric symptoms. Habitual patterns are identifiable, and activity can be inferred.

It should be noted that the movement data was collected over the whole household and includes both PLWD, their carers, and any potential visitors’ movements in the house. This data can be used for trend and pattern analysis by which to identify changes over time or during specific time windows. For example, we have used the in-home movement data in a model to analyse the risk of agitation in PLWD^5^. Fig. 3 illustrates an example of activity patterns of a study participant extracted from the dataset. Figure 3a displays the irregular in-home movements of a PLWD who experienced frequent neuropsychiatric symptoms. Figure 3b illustrates activities of a PLWD with no neuropsychiatric symptoms, where clear habitual patterns are present in daily activities.

**Fig. 4:**
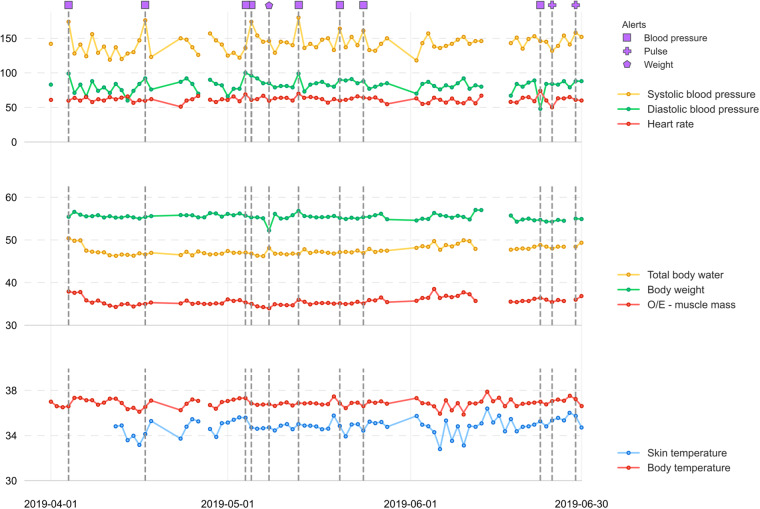
Visualisation of multi-source data for a participant. It is shown the daily physiology data (blood pressure, body weight, temperature) for a participant, aligned with the alerts generated in the dataset.

As an example of multiple sources in the data, in Fig. 4, we combine information from multiple sources of physiology data (e.g., blood pressure, body weight, temperature) for a single participant on a daily basis and display this data aligned with the alerts reported in the dataset. We can see in Fig. 4 that blood pressure alerts were generated when the participant’s blood pressure was higher than the threshold.

## Technical Validation

In order to verify the usability and applicability of the observations and measurements in the dataset for health risk detection or prediction, we have trained and tested a set of classifiers for identifying the risk of *Agitation*. Before training the classifiers, we first aggregated and pre-processed the activity and physiology data according to the following steps:

1. Aggregating location movements by computing statistical attributes of movements at each hour of each day (i.e. sum, mean, maximum, and standard deviation). For example, we obtain four features for describing daily movements in bathroom: “Bathroom_count_sum, Bathroom_count_mean, Bathroom_count_max, Bathroom_count_std”. In this case, “Bathroom_count_mean” indicates the mean of the number of movements in bathroom at each hour of a given day.
2. Aggregating physiology information by taking the maximum values of all measurements in each day. Since most physiological measurements only have one record per day, this step aligns these measurements to one daily figure.
3. Filling in missing values in all numerical features by 0. We intentionally did not apply a data imputation technique at this step to show the effect of missing values in the modelling results. Applying carefully guided imputation methods could improve the results of future experiments.
4. Normalising all numerical features by the min-max normalisation for each participant as: ${x}_{i,p}=\frac{{x}_{i,p}-\min \left({x}_{i,p}\right)}{\max \left({x}_{i,p}\right)-\min \left({x}_{i,p}\right)}$, where *x*_*i,p*_ denotes the subset of the *i*-th feature for participant *p*.
5. Up-sampling positive cases (for samples with validated agitation alerts) in the training set to overcome the class imbalance issue. This is because the positive samples are less than 10% in all training sets of the cross-validation.

**Fig. 5:**
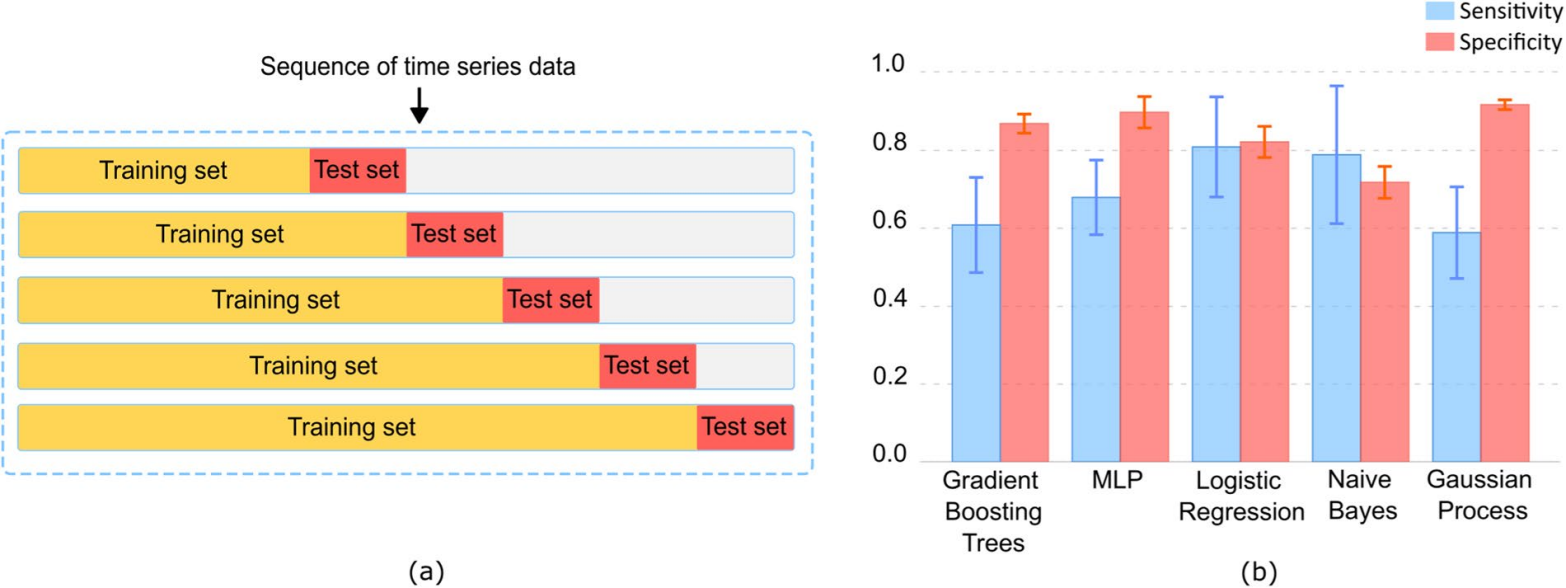
Performance of baseline models for classifying *Agitation* alerts using daily activity and physiology information. (**a**) Demonstration of cross-validation in the experiments with a 5-fold cross-validation as demonstrated and each test set consists of data from a 7-day period. (**b**) Average sensitivity and specificity of the baseline models across 5-fold cross-validation, with error bars indicating the standard deviation for each model.

**Fig. 6:**
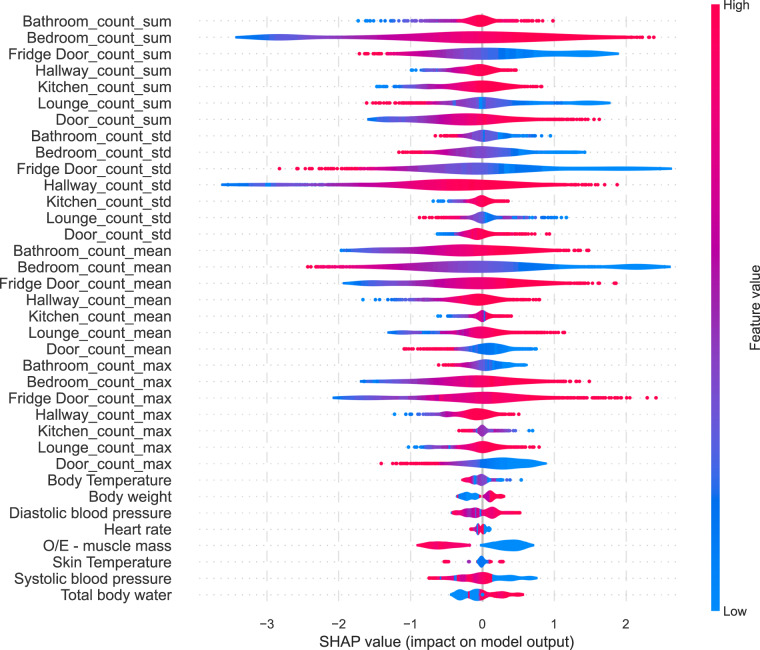
The feature importance learned by the Logistic Regression model. The SHAP value^14^ of each feature represents its impact on the model output regarding a given input. The violin plot illustrates the distribution of SHAP values for each feature, which are estimated by test samples during the cross-validation. The colour indicates whether the raw value of a feature is high or low.

Five baseline models were evaluated, including Gradient Boosting Trees, Multi-Layer Perceptron, Logistic Regression, Naäve Bayes, and Gaussian Process. In our experiments, we applied a 5-fold cross-validation (as shown in Fig. 5a) to evaluate the performance of the baseline models, taking into account the sequential nature of time series data. Figure 5b shows the performance of all baseline models, which demonstrates the potential of developing predictive and analytical models using TIHM dataset^10^ for applications in health and well-being analysis. We also visualise the feature importance metrics learned by the Logistic Regression model in Fig. 6. The SHapley Additive exPlanations (SHAP) value^14^ of each feature represents its impact on the model output regarding a given input. Figure 6 illustrates the distribution of SHAP values for each feature, which are estimated by all test samples during the cross-validation. The colour spectrum in Fig. 6 indicates whether the raw value of a feature is high or low. This helps to verify which features contribute more to the positive or negative predictions.

More advanced methods for feature engineering and data modelling can potentially improve the predictive performance of this experiment by further consideration of the temporal dependencies within the longitudinal data that are not captured in these baseline models. Here we mainly focused on presenting a baseline sample and showcasing the use of the dataset.

## Usage Notes

The TIHM dataset^10^ offers preliminary data to design and validate clinically applicable machine intelligence and decision-support methods for continuous healthcare monitoring. We have provided raw data and guidelines on how to access, visualise, manipulate and predict health-related events within the dataset, available on the Github repository (https://github.com/PBarnaghi/TIHM-Dataset). The Jupyter Notebooks have been developed using Python 3.9.

The dataset is organised in five separate tables stored as separate CSV files, including, Activity, Sleep, Physiology, Labels and Demographics. Data can be cross-referenced across the files. The instructions for loading the data and a set of sample codes for loading and using the dataset are provided in the supplementary code.

## Data Availability

The TIHM dataset is available in the corresponding Zenodo repository^10^ and consists of five separate tables (Activity, Sleep, Physiology, Labels, and Demographics). For further information on the data records, please refer to the README file. The code for the experiments presented in the manuscript is available on the Github repository (https://github.com/PBarnaghi/TIHM-Dataset). The libraries and their versions and dependencies that are used in the code are also provided as a separate configuration file in JSON/YAML format.
